# Recognition of spectrally shaped speech in speech-modulated noise: Effects of age, spectral shape, speech level, and vocoding

**DOI:** 10.1121/10.0017772

**Published:** 2023-04-07

**Authors:** Daniel Fogerty, Jayne B. Ahlstrom, Judy R. Dubno

**Affiliations:** 1 Department of Speech and Hearing Science, University of Illinois Urbana-Champaign, Champaign, Illinois 61820, USA; 2 Department of Otolaryngology-Head and Neck Surgery, Medical University of South Carolina, Charleston, South Carolina 29425, USA dfogerty@illinois.edu, ahlstrjb@musc.edu, dubnojr@musc.edu

## Abstract

This study examined the recognition of spectrally shaped syllables and sentences in speech-modulated noise by younger and older adults. The effect of spectral shaping and speech level on temporal amplitude modulation cues was explored through speech vocoding. Subclinical differences in hearing thresholds in older adults were controlled using threshold matching noise. Older, compared to younger, adults had poorer recognition but similar improvements as the bandwidth of the shaping function increased. Spectral shaping may enhance the sensation level of glimpsed speech, which improves speech recognition in noise, even with mild elevations in hearing thresholds.

## 1. Introduction

Hearing loss associated with age (i.e., presbycusis) is characterized by gradual sloping high-frequency thresholds (ISO, 2017). Spectral shaping, commonly implemented in hearing aid signal processing, involves increasing speech levels to achieve differing amounts of amplification across the speech spectrum, as required by elevated hearing thresholds. Spectral shaping is based on speech intelligibility models that are designed to maximize audibility (ANSI, 1997). Overall, spectral shaping accounts for audibility-related differences in performance between older adults with normal and impaired hearing (Humes, 2007). However, in addition to improving speech audibility, spectral shaping alters the spectral balance between lower and higher frequencies (Fogerty *et al.*, 2015), which may influence how temporal cues are compared and integrated across frequency regions and by different age groups (Fogerty and Humes, 2012). It is, therefore, unclear how spectral shaping may influence speech recognition for younger and older adults independent of improvements in speech audibility.

The amount of spectral alteration applied by spectral shaping to restore audibility can be parameterized in terms of the start frequency (i.e., frequency at which spectral gain begins) and amount of frequency gain (i.e., gain applied to individual frequency bands, determined by speech level and hearing thresholds). Lower start frequencies result in a broader bandwidth for speech amplification, potentially improving audibility. However, lowering the start frequency results in the alteration of larger portions of the spectrum. This may result in perceptual tradeoffs between improved audibility and speech distortion, such as those observed for frequency lowering strategies (e.g., Alexander, 2013). Effects of this spectral distortion may potentially alter phonetic cues. For example, the relative ratio of low-to-high–frequency energy is a potential cue that differentiates stop and fricative consonants (discussed by Chen and Loizou, 2010). In addition, relative differences in formant amplitudes or in spectral tilt can alter vowel perception (e.g., Kiefte *et al.*, 2010). Changes in spectral tilt can also alter contrastive effects that are also known to influence phonetic perception (e.g., Alexander and Kluender, 2008). However, at a sentence level, reductions in spectral tilt, such as that which occurs with Lombard speech, is known to contribute to improvements in sentence recognition (e.g., Lu and Cooke, 2009). This study was specifically designed to assess how these interrelated changes in the spectral shape may affect recognition for syllables and sentences.

In addition to altering the speech spectrum, spectral shaping also results in higher overall speech levels that can reduce, rather than improve, speech recognition under certain circumstances (Amos and Humes, 2007). Relatively high speech levels, which can occur with spectrally shaped speech, result in cochlear nonlinearities that may reduce temporal envelope perception (Dubno *et al.*, 2012). Effects of higher speech levels alone are important to consider when examining recognition of spectrally shaped speech, as well as the possible interaction between altered spectral shape and higher speech levels (Fogerty *et al.*, 2020). However, it is still unclear how various parameters of the spectral shape may affect recognition and how these factors may interact with processing the amplitude modulation of speech (as in Dubno *et al.*, 2012). Toward this end, this study also examined the effect of spectral shaping on the recognition of vocoded speech (i.e., speech with primarily temporal cues). In this way, we assess the extent to which spectral shaping specifically alters the contribution of temporal speech cues to speech recognition. Furthermore, we assess the influence of spectral shaping on syllables, constrained to short temporal modulations of phonetic content, and sentences, which provide longer contexts that also contain slower modulatory rates related to sentence prosody.

We have recently documented that spectral shape, speech level, and hearing thresholds are important factors that explain the speech recognition of older adults with hearing loss, particularly in modulated noise backgrounds (Fogerty *et al.*, 2020). Such modulated background conditions require the listener to glimpse speech within the dips of the masker, which may be constrained by reduced sensation levels (Fogerty *et al.*, 2020, 2021) or affected by the ability to separate sound sources based on modulation cues (e.g., Fogerty *et al.*, 2016; Stone and Moore, 2014). Therefore, the spectral conditions in the current study are tested within a modulated background noise in which recognition may be determined, in part, by “dip listening” and modulation masking.

Thus, the current study was designed to investigate the effects of spectral shape and speech level on speech recognition by younger and older adults. As altered spectral shape and increased speech levels have been known to influence the perceptual processing and resolution of temporal envelope cues, respectively, this study also examined speech with primarily temporal cues through speech vocoding and across different timeframes of speech from syllables to sentences. To minimize the effect of individual differences in hearing thresholds, the current study used a threshold matching noise (TMN) combined with low-pass filtering of the speech. Furthermore, in addition to recognition measures, we document complementary measures of listening effort and a pre-/post-test measure of perceptual adaptation to the novel speech spectrum (see supplementary material for the dataset and analysis of subjective ratings, block completion durations, and pre/post-test sentence recognition).^1^ Through the combination of these factors, the current study detailed if and how spectral shaping, influenced by the start frequency and speech level, may affect speech recognition when audibility factors are controlled.

## 2. Methods

### 2.1 Participants

**Fig. 1.**
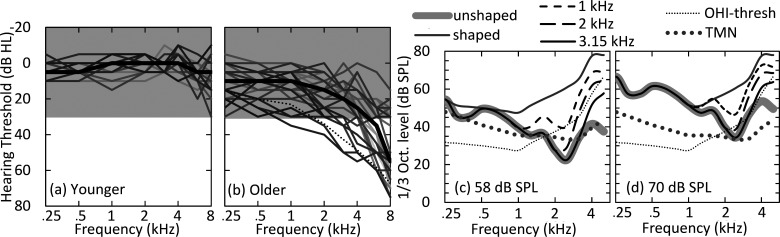
Audiograms for (a) younger and (b) older adults. Bold lines display the median hearing threshold. Shading indicates the region that was masked by a threshold matching noise (TMN). The black dotted line displays the threshold level used for spectral shaping (OHI-thresh) from Fogerty *et al.* (2020). (c) and (d) Long-term average spectra for speech stimuli (matrix sentences) with TMN and OHI-thresh.

Two groups of adults participated in this experiment. Twenty younger adults (15 female, 5 male) were 19–30 years of age [mean = 25.0 years, standard deviation (SD) = 3.1 years]. Younger adults had hearing thresholds ≤ 20 dB hearing level (HL) at octave frequencies from 0.25–8 kHz, except for one participant who had a threshold of 30 dB HL at 8 kHz. Twenty-seven older adults (19 female, 8 male) were 61–84 years of age (mean = 70.5 years, SD = 5.6 years). Inclusion criteria for older adults defined hearing thresholds at or better than 35 dB HL from 0.25–1 kHz, sloping to 55 dB HL at 4 kHz. These criteria were defined based on the mean audiogram for a group of older adults with hearing loss (OHI-thresh) from Fogerty *et al.* (2020) that was used for spectral shaping [see dotted line in Fig. 1(b)]. The majority of older adults had thresholds ≤ 30 dB HL in this frequency range (N = 20). Two older adults had steeper slopes from 1–4 kHz relative to OHI-thresh and were the only participants that had clinically fit hearing aids. Audiograms for the test ear (i.e., the right ear unless the left ear met inclusion criteria better) are displayed in Figs. 1(a) and 1(b). Mini-mental state exam (MMSE) (Folstein *et al.*, 1975) scores for the older adults were at least 27/30.

### 2.2 Experimental design and stimuli

Participants completed closed-set speech recognition testing with two sets of speech materials: nonsense syllables and matrix-style sentences. Each corpus was tested using natural or vocoded speech (tested during different sessions) at two speech levels (58 and 70 dB SPL) and with five spectral shaping conditions [unshaped, three linear spectral shaping functions, and older hearing-impaired (OHI) based shaping]. All testing was completed in a background of speech-modulated noise (SMN). Subclinical differences in speech audibility were reduced using the additional TMN. These conditions are explained below. Perceptual adaptation to spectrally shaped speech was also assessed using sentences during pre- and post-tests for each test session (i.e., natural and vocoded speech).

#### 2.2.1 Stimulus materials

Consonant-vowel (CV) nonsense syllables consisted of 19 consonants /b, p, t, d, k, g, m, n, l, r, w, j, s, z, f, v, ɵ, ʃ, ʧ/ paired with the vowel /a/ that were spoken by a male talker from the University of California Los Angeles version of the Nonsense Syllable Test (NST) (see Dubno and Schaefer, 1992). Matrix sentences were recordings from a male talker (Humes *et al.*, 2014) speaking full sentences in the format ⟨name⟩ ⟨verb⟩ ⟨number⟩ ⟨adjective⟩ ⟨noun⟩. Ten keywords were available for each word position within the sentence (50 total). IEEE sentences (IEEE, 1969; recorded by Loizou, 2007) were used to assess perceptual learning consist of low-context sentences with five keywords per sentence (e.g.,“The *birch canoe slid* on the *smooth planks*”; keywords in italics).

#### 2.2.2 Speech processing

Each set of speech materials (NST, Matrix, and IEEE) was tested as natural speech or with speech vocoding. Natural and vocoded speech were both passed through a bank of 18 1/3-octave analysis filters. The temporal envelope of each analysis band was extracted using the Hilbert transform, low-pass filtered at 16 Hz using a 1st-order Butterworth filter, and recombined with the fine structure of the original speech (natural condition) or noise matching the spectrum of the stimulus file (vocoded condition). Individual bands were then backward filtered by the same analysis bands and summed to create the final stimulus. This processing ensured that the natural and vocoded conditions underwent the same stimulus filtering confined to the same temporal properties. Speech levels, prior to spectral shaping, were calibrated to 58 and 70 dB SPL.

#### 2.2.3 Noise processing

Two types of noise were simultaneously presented with the speech during testing: TMN and SMN. The purpose of the TMN was to minimize subclinical differences in speech audibility between younger and older adults. Following procedures of Dubno *et al.* (2005), the TMN was created by digitally generating a Gaussian noise and adjusting the spectrum at 1/3-octave intervals to achieve masked thresholds at 30 dB HL for the full speech bandwidth. All speech materials were tested within the presence of this TMN. A 5 min TMN was looped continuously throughout the duration of testing and was not spectrally shaped.

All stimuli were also presented in the SMN background. The SMN matched the long-term average speech spectrum of the CV or sentence materials and was amplitude modulated by the temporal envelope of the respective concatenated speech corpus. The temporal envelope was derived using half-wave rectification and low-pass filtered at 16 Hz using a 6th-order Butterworth filter. SMN was presented at a signal-to-noise ratio (SNR) of −5 and 0 dB for natural and vocoded speech, respectively, relative to the root mean squared level of the speech and noise. Different SNRs were selected for natural and vocoded speech to avoid floor and ceiling effects and roughly match performance levels between conditions. A random selection of the SMN began/ended 250 ms from stimulus onset/offset and was ramped by a 20 ms raised cosine ramp.

#### 2.2.4 Spectral shaping of speech and SMN

Five spectral shaping conditions were examined. Speech and SMN were unshaped, shaped to fit the hearing loss (HL-shape) from OHI-thresh, or linearly shaped across 1/3-octave frequency bands from the start frequency (1, 2, or 3.15) with increasing gain up to an 8 kHz target. HL-shape used procedures from Fogerty *et al.* (2020) to apply gain individually to 1/3-octave bands to achieve band sensation levels of at least 20 dB (except for two older adults who only obtained 15 dB sensation levels from 1–3.15 kHz) and was only tested at 70 dB SPL. Pre-/post-test IEEE sentences were only processed according to HL-shape. To control for reduced audibility at higher frequencies in the older participants, the bandwidth of the speech and SMN for all conditions was reduced by low-pass filtering at 5.6 kHz. Figures 1(c) and 1(d) display the effect of spectral shaping on the speech spectrum across these five conditions. This resulted in a total of 18 experimental conditions [2 speech materials (syllables, sentences) × 2 speech levels (58 and 70 dB SPL) × 4 spectral conditions (unshaped and three start frequencies) + 2 HL-shape conditions (syllables and sentences each at 70 dB SPL] for each of two speech conditions (natural and vocoded).

### 2.3 Procedures

Participants were tested in a sound-attenuating booth seated in front of a touch screen monitor running a custom closed-set response interface operating in matlab (MathWorks, Natick, MA). The participants listened to stimuli at a sampling rate of 48.8 kHz presented through a Tucker-Davis Technologies (Alachua, FL) System III digital-to-analog processer (RX6). The TMN was played out of a Lynx (San Jose, CA) L22 soundcard and added to the speech + SMN mixture using a signal mixer (SM3) and the combined signal was passed through a headphone buffer (HB6) and delivered to the test ear of Sennheiser (Wedemark, Germany) HDA 200 headphones.

**Fig. 2.**
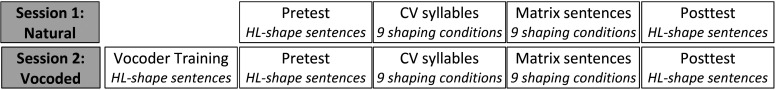
Block diagram of the experimental procedure.

A block diagram of the experimental procedure is displayed in Fig. 2. Testing was completed in two sessions for natural and vocoded speech, in that order. Within a test session, testing proceeded on the order of pretest, CV syllables, matrix sentences, and post-test. Pre-/post-testing consisted of open-set testing of 10 IEEE sentences presented in SMN. Participants were required to repeat the sentence aloud. For the vocoded session, an additional training block was presented first to help familiarize participants to the novel stimulus. Vocoded training consisted of brief exposure in quiet and SMN. Each block consisted of an initial set of five IEEE vocoded sentences that participants repeated aloud. After each response, auditory feedback was provided for each stimulus by playing the undistorted stimulus followed by a repetition of the vocoded stimulus (similar to Davis *et al.*, 2005).

During experimental testing for syllables and sentences, participants responded *via* button press on the touch screen. Stimulus presentation was self-paced. Testing was blocked by speech level, which participants received in a random order. A familiarization consisting of four trials, processed in different spectral conditions, was presented prior to each block. Within a speech level block, the order of the spectral shaping conditions was randomized. Following all testing, the HL-shape condition was always presented last as comparison. For syllable testing, all 19 syllables were tested twice within an experimental condition (38 syllable targets), resulting in 342 trials for each speech type [38 syllable targets × (2 speech levels × 4 shaping conditions + 1 HL-shape condition)]. For sentence testing, eight sentences were selected randomly (without replacement) for each condition and processed online, resulting in 40 keywords per condition. This resulted in 360 keywords per speech type.

Following each condition, participants completed subjective ratings in matlab using a slider on a visual analogue scale corresponding to the performance and effort subscales of the NASA-TLX (Hart and Staveland, 1988). The time to complete each condition was also recorded. These additional data are complementary to the primary experimental hypotheses and will not be discussed further (See supplementary material for the full dataset).^1^

## 3. Results

### 3.1 Experimental conditions

**Fig. 3.**
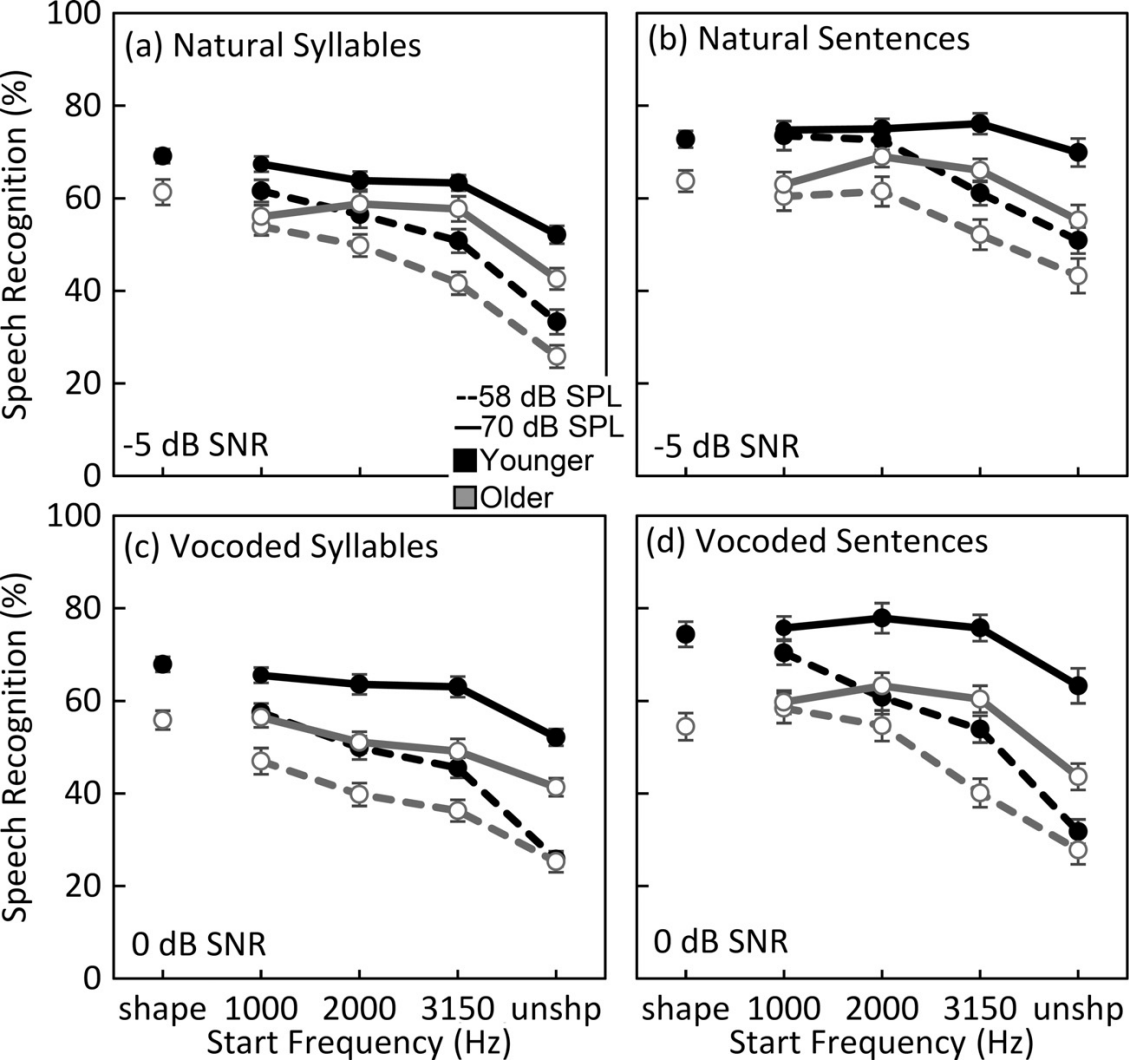
Recognition of (a) and (b) natural and (c) and (d) vocoded speech for the five spectral shape conditions for younger (black) and older (gray) adults. Error bars indicate ± 1 standard error of the mean.

Speech recognition scores for syllables and keywords are plotted in Fig. 3 as a function of the start frequency. Full-spectrum shaping based on the OHI thresholds is presented as the left-most data point, and unshaped speech is represented as the right-most data point (equivalent to the extreme start frequency). Thus, these figures can be read from left to right as decreasing the bandwidth of the shaped spectrum. From the figure, a general trend is observed across speech materials and natural and vocoded speech, with decreasing keyword recognition as the shaped bandwidth becomes narrower. Effects of age and speech level also are apparent, with younger adults and the higher speech level resulting in better performance.

These observations were confirmed by a series of mixed model analyses of variance with group as the between-subject variable and speech level and start frequency as the within-subjects variable (Table 1). The HL-shape condition was not included in this analysis as it did not involve a linear function across frequency. Results of these analyses are provided in Table 1. Main effects of level, start frequency, and group were observed, with the interaction between level and start frequency reflecting the steeper decline in performance with increasing start frequency for the lower speech level. This indicates that there was a greater benefit of spectral shaping for the lower speech level, which corresponded to a lower sensation level (in the presence of TMN). Significant group interactions were not observed indicating similar effects for both age groups. Effect sizes were generally larger for vocoded compared to natural speech.

**Table 1.** Results for the analysis of variance test

Factor	F(df)	*p*	η_p_^2^	F(df)	*p*	η_p_^2^
	*Natural syllables*			*Natural sentences*		
Level (L)	77.3 (1,45)	<.001	.63	56.8 (1,45)	<.001	.56
Start Freq (SF)	108.1 (3,135)	<.001	.71	41.4 (3,135)	<.001	.48
L × SF	17.8 (3,135)	<.001	.28	10.6 (3,135)	<.001	.19
Group	10.0 (1,45)	.003	.18	11.6 (1,45)	.001	.21
	*2.1 (1,44)* ^a^	*.150*	*n.s.*	*5.2 (1,44)*	*.028*	*.11*
	*Vocoded syllables*			*Vocoded sentences*		
Level (L)	214.6 (1,45)	<.001	.83	94.9 (1,45)	<.001	.68
Start Freq (SF)	110.2 (3,135)	<.001	.71	122.7 (3,135)	<.001	.73
L × SF	11.7 (3,135)	<.001	.21	25.2 (3,135)	<.001	.36
Group	15.3 (1,45)	<.001	.25	14.7 (1,45)	<.001	.25
	*5.2 (1,44)*	*.027*	*.11*	*5.0 (1,44)*	*.031*	*.11*

^a^ Italics denote results of group when controlling for four-frequency pure-tone average (PTA4).

The effect of group generally remained significant if participants with four-frequency pure-tone averages (PTA4; 0.5, 1, 2, 4 kHz) greater than 30 dB HL were excluded from the analysis (η_p_^2^ effect sizes ranged from 0.2–0.3) or if PTA4 (younger: M = 1.4 dB, SD = 3.6 dB; older: M = 16.9 dB, SD = 8.7 dB) was entered as a covariate in the analysis (italics in Table 1). These results suggest that the group effect is associated primarily with age-related differences in speech recognition, rather than differences in hearing thresholds between the groups that were largely controlled through the use of TMN and low-pass filtering the speech.

Listeners' self-ratings were consistent with these patterns (see supplementary materials for the self-ratings),^1^ and condition durations were longer for the older than younger adults. The pre-/post-test measure demonstrated rapid perceptual adaptation for younger and older adults to spectrally shaped speech for natural but not vocoded sentences, which was not correlated with recognition.

### 3.2 Comparison to OHI spectral shaping

As a comparison to more realistic spectral shaping, paired-samples t-tests were conducted between the HL-shape condition and the 1 kHz start frequency (two left-most data points in Fig. 3). No comparisons were significant at an alpha level of 0.05 for either group after Bonferroni correction for multiple comparisons. This indicates that speech recognition for the spectral shaping conditions tested here was similar to that for hearing-aid–type processing that would more commonly be provided to listeners with hearing loss.

## 4. Discussion

Overall, these results demonstrate that listeners obtain benefit for spectrally shaped speech when audibility is limited by TMN (intended to model elevated hearing thresholds and resulting differences in speech audibility). Similar effects were observed for CV syllables and sentences in SMN, indicating a general benefit of spectral shaping on speech recognition. These results indicate that, when compared to unshaped speech, spectral shaping results in improvements in speech recognition, particularly at lower speech levels. This benefit includes recognition based primarily on amplitude modulation cues of speech (i.e., vocoded condition). Therefore, the effects of spectral shaping and higher speech level did not appreciably affect the contribution of amplitude modulation cues to speech recognition in this study (in contrast to Dubno *et al.*, 2012).

Poorer recognition was observed with higher start frequencies. At least two factors could explain this result. Increasing the start frequency of spectral shaping has the effect of increasing the slope of the gain function, leading to more rapid changes in speech gain with increasing frequency compared to lower start frequencies. This steep slope to the gain function could result in a sharper level difference between low and high frequencies (i.e., greater contrastive effects) (Alexander and Kluender, 2008). Alternatively, the higher start frequency also reduces the bandwidth of speech amplification, resulting in reduced sensation levels within the dips of the SMN relative to the hearing threshold (determined primarily by the TMN). The unshaped spectral condition is informative in dissociating these two possibilities as it provided reduced sensation levels without spectral alterations. Recognition was the poorest in this unshaped condition. This suggests that the declining recognition with increased start frequency was due to the second factor—reduced sensation level during speech glimpses within the modulated masker. This is similar to the effect of Lombard speech, which naturally increases high-frequency energy, resulting in better speech glimpsing in noise (Lu and Cooke, 2009). Importantly, the HL-shape condition, which most reflects clinical hearing-aid prescriptions among the conditions tested here, resulted in the best recognition across all conditions. This condition adequately restored audibility across the spectrum (within 15–20 dB sensation level) for all older adults. Of course, here we used a standard spectral shape that “over-fit” (i.e., provided ≥ 20 dB SL) for most listeners, whereas the clinical approach would be fit to individual hearing thresholds.

This interpretation of improved glimpse sensation levels is consistent with our previous findings that suggest that reduced masking release in modulated maskers for older adults with hearing loss is due, at least in part, to reduced sensation levels during the dips of the masker (Fogerty *et al.*, 2020). Importantly, the sensation level benefits obtained by spectral shaping, at least in this study, did not appear to interact with moderate speech levels or with the temporal modulation properties of speech. It may be that higher speech levels than tested here are required to observe distortions of temporal envelope perception that could affect speech recognition (i.e., Dubno *et al.*, 2012). However, Dubno *et al.* (2012) observed declining speech recognition from 60–85 dB SPL, which covers the range of 58 dB SPL for the low-level unshaped speech to 82 dB SPL for shaped speech in this study. It may be that the reduction in sensation levels due to the TMN in the current study explains the continued benefit of spectral shaping and accompanying increase in speech level in this study, even with potential temporal distortions.

Younger and older adults differed in their overall speech recognition yet obtained similar patterns across spectral conditions. Age group differences in this study do not appear to be a result of differences in peripheral hearing sensitivity. First, TMN was provided to account for subclinical differences in speech audibility. Second, stimuli were low-pass filtered at 5.6 kHz to account for differences in high-frequency hearing. Third, neither excluding older adults with the poorest thresholds, nor controlling for PTA4 within the analysis, explained group differences. These results suggest an age-related, rather than hearing-loss–related, source of group differences. This is consistent with other explanations of age-related differences in speech recognition in noise (e.g., Bologna *et al.*, 2018) that remain after accounting for differences in hearing thresholds (see Humes, 2007), potentially due to differences in central auditory or cognitive abilities (e.g., Committee on Hearing, Bioacoustics and Biomechanics (CHABA), 1988).

Overall, the results of this study indicate that, when speech audibility is reduced for younger or older adults due to even minor elevations in hearing thresholds (i.e., 30 dB HL), spectral shaping facilitates speech recognition by enhancing the sensation level of speech glimpses. (This was also true for the seven older adults with hearing losses that exceeded 30 dB HL in at least one frequency.) Significantly, such minor elevations in hearing threshold would typically not result in candidacy for hearing aid prescriptive fittings, yet significant benefits in recognition could be gained with some spectral shaping (see also Roup *et al.*, 2018). Additional research is needed to identify the potential role for over-the-counter and self-fitting hearable technology to fill this gap in treatment. The greatest benefits in recognition were obtained for spectral shaping functions that accounted for the full bandwidth of reductions in audibility, even when these conditions resulted in moderately higher speech levels that have been associated with declines in temporal envelope processing. These results indicate that enhancement of sensation level of speech glimpses is essential for maximizing speech recognition in temporally modulated backgrounds.

## Footnotes

^1^
See supplementary material at https://www.scitation.org/doi/suppl/10.1121/10.0017772 for the dataset and analysis of subjective ratings, block completion durations, and pre/post-test sentence recognition.
